# The Powdering Process with a Set of Ceramic Mills for Green Tea Promoted Catechin Extraction and the ROS Inhibition Effect

**DOI:** 10.3390/molecules21040474

**Published:** 2016-04-11

**Authors:** Kouki Fujioka, Takeo Iwamoto, Hidekazu Shima, Keiko Tomaru, Hideki Saito, Masaki Ohtsuka, Akihiro Yoshidome, Yuri Kawamura, Yoshinobu Manome

**Affiliations:** 1 Division of Molecular Cell Biology, Core Research Facilities for Basic Science, The Jikei University School of Medicine, Tokyo 105-8461, Japan; tmiwamoto@jikei.ac.jp (T.I.); tomaru@jikei.ac.jp (K.T.); h-saito@jikei.ac.jp (H.S.); manome@jikei.ac.jp (Y.M.); 2 Health and Environment Systems Division, Consumer Electronics Company, Sharp Corporation, Osaka 581-8585, Japan; shima.hidekazu@sharp.co.jp (H.S.); ohtsuka.masaki@sharp.co.jp (M.O.); yoshidome.akihiro@sharp.co.jp (A.Y.); tynkerbell1218@hotmail.com (Y.K.)

**Keywords:** green tea, matcha, catechin, polyphenol, powdering process, mill, ROS

## Abstract

For serving green tea, there are two prominent methods: steeping the leaf or the powdered leaf (matcha style) in hot water. The purpose of the present study was to reveal chemical and functional differences before and after the powdering process of green tea leaf, since powdered green tea may contribute to expanding the functionality because of the different ingesting style. In this study, we revealed that the powdering process with a ceramic mill and stirring in hot water increased the average extracted concentration of epigallocatechin gallate (EGCG) by more than three times compared with that in leaf tea using high-performance liquid chromatography (HPLC) and liquid chromatography–tandem mass Spectrometry (LC-MS/MS) analyses. Moreover, powdered green tea has a higher inhibition effect of reactive oxygen species (ROS) production *in vitro* compared with the same amount of leaf tea. Our data suggest that powdered green tea might have a different function from leaf tea due to the higher catechin contents and particles.

## 1. Introduction

The health benefits of green tea consumption have been reported to include: cancer inhibition [1,2], allergy relief effects [3], cognitive dysfunction [4], and preventive effects on metabolic syndrome [5,6]. In a large epidemiological study, which was based on a follow-up investigation of 82,369 Japanese people for 13 years, green tea consumption also showed positive effects such as lowering the risk of cardiovascular diseases and stroke [7]. Moreover, another follow-up study of 90,914 Japanese people for 18.7 years suggested that the consumption of green tea reduces the risk of total mortality and three leading causes of death: heart disease, cerebrovascular disease, and respiratory disease [8]. This evidence of health benefits should attract the attention of health-conscious people.

There are two prominent methods for serving green tea. The general serving method consists of steeping the leaves in hot water and filtering them through a tea strainer. In contrast, in traditional Japanese tea ceremony, fine powdered green tealeaves (matcha) are foamed with a tea whisk in hot water. Ingestion of matcha likely allows for the introduction of a large amount of tealeaf components into our body.

Matcha is typically produced from shade-grown green tealeaves that are steamed, dried, and then ground with a set of millstones. According to Sawamura *et al.* [9], it is unknown when millstones came into use. However, since ancient documents have reported the use of millstones for tea leaves in the 11th century in China [9,10] and in the 14th century in Japan [9,11], it is likely that the ingestion of powdered green tealeaves may have been a habit for a long time.

Major components of matcha [12] are catechins, which are known for their anti-oxidant activity [13], amino acids, and saponins, which contribute to the foaming property of matcha [14]. Using high-performance liquid chromatography (HPLC) analysis, Saijo *et al.* [15] showed that catechins make up 4.92% of the dry weight of matcha. In addition, liquid chromatography–mass spectrometry (LC-MS) quantification of catechins in commercially available green tea by Goto *et al.* [16] showed that epicatechin (EC), epigallocatechin (EGC), epicatechin gallate (ECG), and epigallocatechin gallate (EGCG) were the four main types of catechin present in matcha with an average content of 11.19%, while the caffeine content was 3.77%. Moreover, EGCG has predominant functions as a highly anti-oxidant compound [17,18] among green tea catechins.

Studies on the physical properties of matcha have revealed differences in particle sizes, shapes, and foaming properties as the result of diverse powdering methods. Onishi *et al.* [19] reported matcha particles of several micrometers by using electron microscopy, while Sawamura *et al.* reported median diameters of 15–20 µm (millstone and ball mill), or ≤5 µm (jet mill) using laser diffraction analysis [20,21].

Although there are many reports on the chemical and physical properties of powdered green tea, changes in its properties before and after powdering process using the same tealeaves have not been revealed. Since changes in the powdering process may alter the chemical components and functionality of the green tea, thus contributing to expand its health-promoting benefits, we investigated the differences in physical property, catechin concentration, and reactive oxygen species (ROS) inhibitory effect of green tea prepared from whole leaves or powdered leaves.

## 2. Results

### 2.1. Particle Appearance in Different Powdering Methods

**Figure 1:**
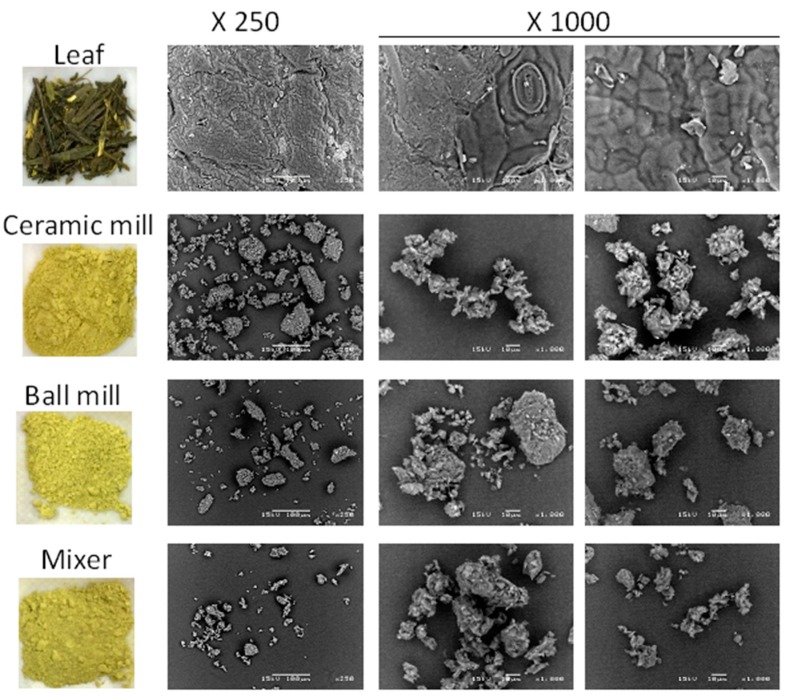
Appearance of green tea leaf and powder produced with different powdering method. Left panel (color) shows the photograph of green leaf and the powders produced with indicated powdering process. Right panel (monochrome) shows the images of leaf and the powders using the scanning electron microscope at indicated magnification.

The physical properties of whole green tealeaves and those powdered using three different methods were evaluated by electron microscopy (Figure 1). In the case of the tealeaf, the tissue structure of the plant was partially maintained, and small granular particles of 1–20 μm were observed around the tealeaves. In contrast, when powdered with a ceramic mill, ball mill, or mixer, many leaves were miniaturized into particles of less than 100 µm. Furthermore, the use of a ceramic mill, which is characterized by a strong shearing force [20], resulted in particles with torn shapes in comparison to other methods.

**Figure 2:**
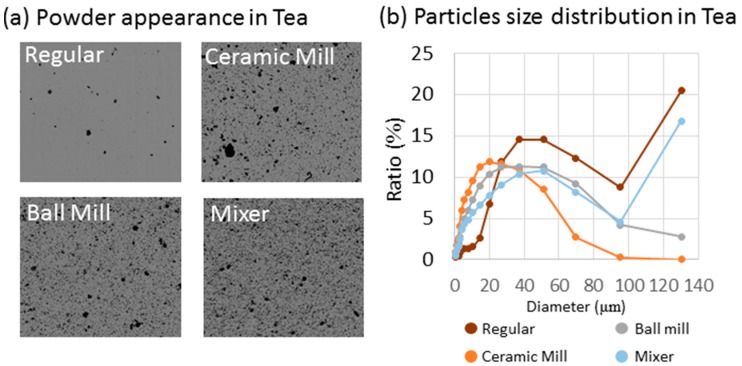
Particle appearance in the green tea liquid and size distribution of tea particles: (**a**) multifocal microscope images of particles in regular tea and the powdered tea produced by indicated powdering process; and (**b**) particle size distributions of the teas measured with laser diffraction particle size analyzer. Regular: Regular leaf tea.

Next, the whole and powdered tealeaves were brewed in hot water and observed with multifocal optical microscope in an automated cell counter. Many black particles were observed when powdered with the ceramic mill, ball mill, and mixer (Figure 2a). In contrast, regular tea from steeped tealeaves showed fewer black particles, although faint grey micro particles were also observed. The number of particles in a 4-mm^2^ field (Figure 2a) was determined with image analysis software (WinRoof 2013) as 2910–3014 for regular tea, including faint gray micro particles; 14,760–15,767 for the ceramic mill; 16,135–18,583 for the ball mill; and 13,374–18,090 for the mixer. In addition, the particle size distribution showed that median particle size was 49.20 µm for normal green tea, 15.01 µm for the leaves powdered with the ceramic mill, 23.03 µm for those treated with the ball mill, and 31.85 µm for those processed with the mixer (Figure 2b).

### 2.2. Catechin Contents of Powdered Tea and Leaf Tea

Based on the particle size differences and characteristic shapes, we compared the release of components from the leaf tea (Leaf) and the powdered tea produced with the ceramic mill (Powder).

The concentration of polyphenol, which is the major green tea component, was measured using Folin–Denis method (Table 1). In this experiment, three types of tea were prepared: (1) the regular brewing method (Regular); (2) brewing powder with hot water and stirring for one minute with a stirrer to mimic a tea ceremony method (Powder and Strong Powder); and (3) brewing the leaves with hot water and stirring for one minute as the control (Leaf and Strong Leaf). Concentration of total polyphenol was higher in the Strong Powder and Strong Leaf tea than in the Regular tea. Moreover, compared to the Leaf teas, the Powder teas had a higher total polyphenol concentration at same leaf/powder contents. These results indicate that polyphenol extraction is accelerated by the grinding process with the ceramic mill and the stirring process at brewing.

**Figure 3:**
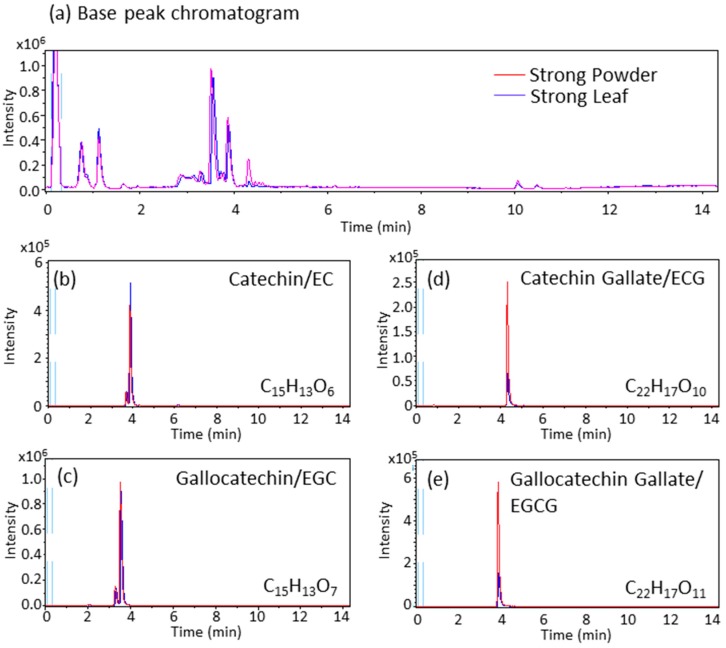
Differences of catechins between Powder and Leaf green tea in liquid chromatography–mass spectrometry (LC-MS) chromatogram (negative mode, representative data in three measurements): base peak chromatogram (**a**); and extracted ion chromatogram of indicated catechins ((**b**)–(**e**)).

The concentrations of the four major catechins (EC, EGC, ECG, and EGCG) and caffeine component in the teas were measured using HPLC (Table 1). ECG, EGCG and caffeine were detected at significantly higher concentrations in Strong Powder tea. In addition, both Powder teas showed the significantly higher concentrations of EGCG and caffeine and showed the tendency of higher concentration of ECG than Leaf teas at the same contents of powder/leaf. These results indicate that powdering process with the ceramic mill and stirring process might more efficiently extract ECG and EGCG, which obtains galloyl moiety (Table 1). Liquid chromatography–tandem mass spectrometry (LC-MS/MS) analysis of Strong Powder tea and Strong Leaf tea showed negativemolecular ion [M − H]^−^ with *m*/*z* 289.07, 305.07, 441.08, and 457.08, indicating catechin/EC, gallocatechin/EGC, catechin gallate/ECG, and gallocatechin gallate/EGCG, respectively (Figure 3 and Table 2). These data suggest that Strong Powder tea contains significantly higher amounts of EGC, ECG, and EGCG, and a lower amount of EC compared to Strong Leaf tea (*p* < 0.05).

### 2.3. Effect of Powdered Tea and Leaf Tea on ROS

**Figure 4:**
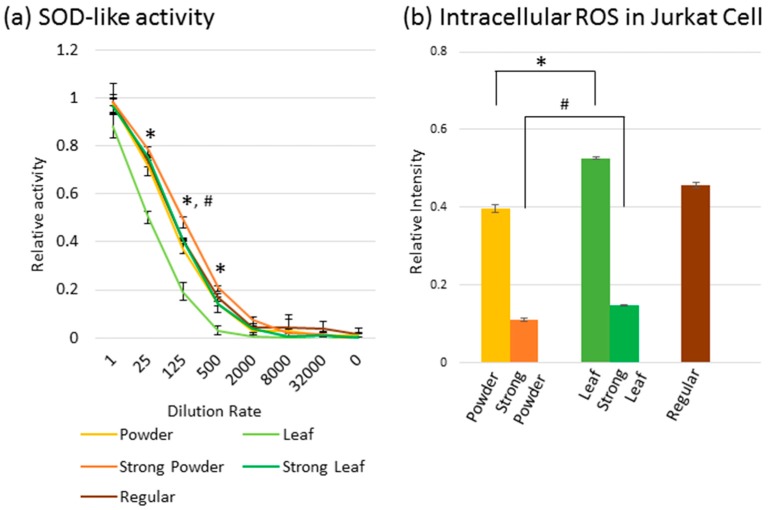
Differences in reactive oxygen species (ROS) inhibitory effects of green teas *in vitro*: (**a**) superoxide dismutase (SOD)-like activity of green teas at indicated dilution rate; and (**b**) intracellular ROS intensity in Jurkat cell line added with green teas (1/500 dilution). * and # indicate differences between Powder *vs.* Leaf tea and Strong Powder *vs.* Strong Leaf tea at 5% significant level, respectively (Bonferroni/Dunn method).

To appraise whether the physical and chemical differences observed in leaves and powdered tealeaves might have been coupled with functional differences, we examined the inhibitory effect on ROS *in vitro* (Figure 4). When the same amount of powder and leaves were compared, the superoxide dismutase (SOD)-like activity, which counteracts the ROS effect, showed higher tendency in the Powder tea and Strong Powder tea than in the leaf teas (Figure 4a). Consistently, ROS inhibition effect in the Jurkat human leukemia cell line was higher for both powder teas compared to the same amount of leaf teas (Figure 4b).

## 3. Discussion

This study aimed to investigate the differences in composition and function between leaf tea and powdered tea. We revealed the physical and chemical component changes after the powdering process, which may be the basis of different activity *in vitro*.

### 3.1. Physical Properties of the Powdered Green Tea

Comparison of the powdering process revealed that the ceramic mill produced the smallest tea particles. A previous report from Sawamura *et al.* [20] indicated that a set of millstones had higher shearing force and produced lower roundness of particles than a jet mill. In line with Sawamura’s study, our results, using Scanning Electron Microscope (SEM) images of green tea powder derived by a ceramic mill, confirm the predominant presence of torn structures rather than other methods (Figure 1). Additionally, the ceramic mill and the millstone method produced particles of similar sizes in the report (15.01 µm *vs.* 15–20 µm [20,21]). Therefore, tea powder with the ceramic mill may show a similar property of tea powder produced with the millstones in the historical grinding method.

### 3.2. Catechin and Caffeine Contents of the Powdered Green Tea

The powdering process increased the catechin contents, especially EGCG, in the green tea. There are few reports comparing the amount of catechin in the tea liquid before and after the powdering process, since general studies compare leaf teas and powdered teas from different brands. In a previous study [22], leaves powdering increased the intake of catechins from green tea (*Benifuuki*) in rats in comparison with leaf infusion. In line with this study, our data suggest that the increase of the catechin extraction due to powdering process (Table 1) may enhance its corresponding intake in rats.

Shishikura *et al.* [23] showed that EGCG and caffeine were highly contained in powdered tea (EGCG: 183.2 ± 19.8 mg and caffeine 138.1 ± 17.7 mg on average in 600-mL *Uji-matt-cha* and *Gyokuro-cha*) after 1-min brewing. On the other hand, leaf tea contained 154.6 ± 17.2 mg EGCG and 121.4 ± 4.6 mg caffeine in 600-mL *Sayama-cha* [23]. Compared to other reports, which used organic solvents or longer brewing time for catechin extraction from tealeaves, Shishikura’s report addressed brewing styles relevant to dietary consumption such as the use of boiling water for brewing and shorter brewing times (1 and 5 min). We used hot water for brewing, a 1-min brewing time, and a stirrer, because these conditions were similar to those in a tea ceremony. According to our data (Table 1), an estimated 600-mL of Powder teas contained 126.4–369.1 mg EGCG and 72.9–187.1 mg caffeine, while an estimated 600-mL of Leaf teas contained 34.7–120.1 mg EGCG and 46.7–146.4 mg caffeine. LC-MS/MS data also indicated the higher contents of EGCG (Figure 3 and Table 2). These data suggest that the powdering process promoted the extraction of EGCG and caffeine.

In several clinical studies, the effects of green tea or green tea extract on weight management were investigated. Thielecke *et al.* [6] reviewed the clinical studies focused on a catechin intake of 140.8–1206.9 mg/day, EGCG intake of 100–595.8 mg/day, and caffeine intake of 27–236.7 mg/day. Among 12 such studies, nine studies showed a tendency for weight, fat mass, or Body Mass Index (BMI) to decrease, while two studies showed no change or a tendency for these factors to increase, and one study demonstrated the result of caffeine consumption dependency. Recent studies suggested that the consumption of both caffeine and catechin affected body weight management [24,25]. Moreover, EGCG are known to reduce cholesterol solubility in bile acid micelles, which are related to fat absorption, due to interaction of bile salt [26] and phosphatidylcholine [27]. Thus, powdered green teas may have potential weight management benefits owing to higher extraction efficacy of catechin, especially EGCG, and caffeine contents, although we should pay attention to acceptable daily intake.

As for the intake of polyphenols, most dietary polyphenols are known to be metabolized in phase II reaction (*O*-methylated, sulfated, glucuronide conjugates, *etc.*) or metabolized/degraded by microbiota [28,29]. Stalmach *et al.* [30] investigated the absorption, metabolism, and excretion of Choladi green tea flavan-3-ols in human using LC-MS system. They detected the 15 metabolites of the flavan-3-ols in urine and the 10 metabolites and intact forms of EGCG and ECG in plasma. Since galloyl moiety of EGCG and ECG was thought to interfere with the metabolism, intact forms of EGCG and ECG were detected in plasma [28]. After ingestion of the 500-mL Choladi green tea containing 230 ± 6 μmol EGCG and 49 ± 1 μmol ECG, the C_max_ (maximum concentration) in plasma of EGCG and ECG were 55 ± 12 nM and 25 ± 3.0 nM, respectively [30]. In our study, Powder green teas contained higher concentration of EGCG and ECG, which obtain galloyl moiety, than Leaf teas at the same powder/leaf contents (Table 1). Since an estimated 500-mL of Powdered green teas contained 230–671 μmol EGCG and 40–111μmol ECG (calculated from Table 1), ingestion of Strong Powder tea may contribute to the higher C_max_ or AUC (area under the concentration-time curve) of EGCG and ECG than that of Choladi green tea, while Powder tea may contribute to the similar results of Choladi green tea due to similar concentration of EGCG and ECG. Moreover, particles in the powdered teas may be affected by the digestion in the gastrointestinal tract. Therefore, investigation of bioavailability of powered teas will contribute to revealing the novel function of green tea.

### 3.3. Effect on ROS Activity

Both Powder teas showed the tendency of higher inhibitory effect on ROS than the same amount of Leaf teas. Catechins (EC, ECG, EGC, and EGCG) are known to be antioxidant compounds. Among the catechins, the anti-oxidant activity was higher for EGCG than for EGC, ECG, and EC [13,17,31,32]. Since the powdering process increased the EGCG extraction by more than three times compared with that in Leaf teas (Table 1), higher anti-oxidant activity was expected. Accordingly, both Powder teas showed the tendency of higher SOD-like activity and inhibition of intracellular ROS compared to the same amount of Leaf teas (Figure 4a,b).

On the other hand, Strong Leaf tea has a higher inhibition activity of intracellular ROS than Powder tea, although both teas contained a similar amount of EGCG (200.1 *vs.* 210.6 μg/mL) and ECG (33.3 *vs.* 35.7 μg/mL) and showed similar SOD-like activity (Figure 4a,b, and Table 1). Therefore, the difference of shapes (leaf and powder) or other catechin contents may have an effect on intracellular ROS. Further study is needed to reveal the ROS inhibitory mechanism of powdered tea.

## 4. Materials and Methods

### 4.1. Tea Preparation and Powdering Process

**Figure 5:**
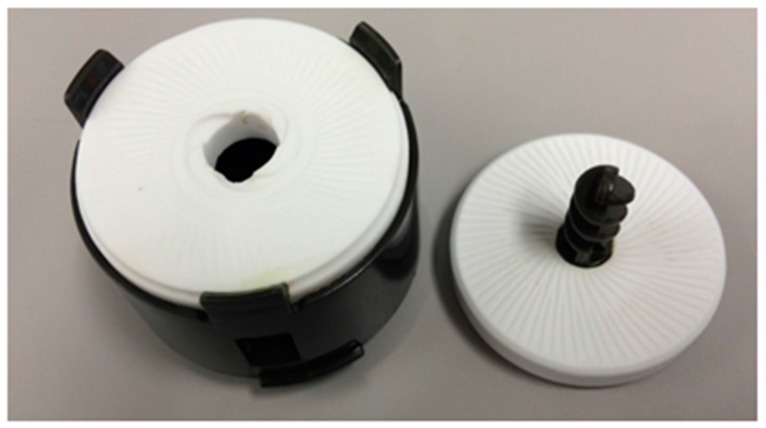
A set of ceramic mill set in a tea maker.

Green tea leaves (second picked tea leaves) were purchased from a tea specialty shop in Tokyo, Japan. For powdering process of green tea leaves, we used a set of ceramic mills (Figure 5) in a tea maker, Healsio Ocha Presso (TE-GS10A, Sharp, Osaka, Japan), a ball mill with an alumina ball, and a mixer with stainless cutter for cooking use for 3–4 min (mill-contact time of tea leaf was approximately 30 s), 420 min, and 5 min, respectively.

Because Shishikura *et al.* [23] mentioned that most individuals in their laboratory (*n* = 34) spend less than 1 min preparing a cup of tea in their paper, we adopted a 1-min brewing time for green tea preparation. Then hot tap water after boiling (60 °C, 80 mL) was added to the tea powder or leaf (0.36 g or 0.96 g) in a conical flask and stirred for 1 min with a magnetic stirrer (SW-RS077, Nisshin Rika, Tokyo, Japan). As for regular tea, hot tap water after boiling (60 °C, 80 mL) was added to tealeaf (2.0 g) in a conical flask and placed for 1 min (no stirring). The aim of regular-tea investigation was the comparison with the general green tea, which we drink daily. After being placed for 1-min on ice, all the tea solutions were transferred into a light-shielding glass bottle using a 10-mL pipet and refrigerated at −20 °C until examination. For investigating the particle distribution (Section 4.4.), the regular tea was filtered with a tea strainer after brewing instead of 10-mL pipet transfer.

### 4.2. Chemicals

(−)-Epigallocatechin (98%), (−)-Epicattechin (98%), Caffeine anhydrous (98.5%), Phosphoric Acid, Ortho (85%), Acetonitrile (99.8%, used for HPLC analysis in this paper) were purchased from Nacalai tesque, Kyoto, Japan. (+)-Catechin Hydrate (>95.0%), (−)-Epicatechin Gallate (>98.0%), (−)-Epigallocatechin Gallate Hydrate (>98%), tert-butyl hydroperoxide (70% in Water) were purchased from Tokyo Chemical Industry, Tokyo, Japan. Acetonitrile for High Performance Liquid Chromatography (99.8+%, used for LC-MS/MS analysis in this paper) and Sodium Carbonate (99.8+%) were purchased from Wako Pure Chemical Industries, Tokyo, Japan. Folin–Denis’ reagent and 2’,7’-dichlorofluorescein diacetate (≥97%, DCFDA) was purchased from Merck KGaA (Sigma-Aldrich), Darmstadt, Germany. Anmonium formate solution, and Formic acid-Acetonitrile were purchased from Kanto Kagaku, Tokyo, Japan. Sodium hexametaphosphate was purchased from Mitejima Chemical, Osaka, Japan. SOD Assay Kit-WST was purchased from Dojindo Molecular Technologies, Kumamoto, Japan.

### 4.3. Observation of Tea Powders and Leaves with a SEM and a Multifocal Optical Microscope

For the observation with an electron microscope, tea powders and leaves were scattered on a carbon tape, followed by blowing the excess powders or leaves. Then powders and leaves were observed with SEM (JSM-5800LV, JEOL, Tokyo, Japan) using accelerating voltage of 15 kV at magnification of 250 and 1000 times. For the multifocal optical observation, 10 µL of tea was injected into a plastic slide and then images were recorded with an automated cell counter (TC20, Biorad, CA, USA). The optical images were analyzed with an image analysis software (WinRoof 2013, Mitani Corporation, Tokyo, Japan) for counting the particles in the picture (around 4 mm^2^).

### 4.4. Size Distribution in Tea Liquid

The size distribution of tea powders was measured with laser diffraction particle size analyzer (SALD 1100, Shimadzu Corporation, Kyoto, Japan) after dispersion in 1% sodium hexametaphosphate in water. For the measurement of particles size in the regular tea, tea liquid after filtration with a tea strainer (Section 4.1.) was used for exclusion of large tealeaves.

### 4.5. Catechin and Caffeine Analysis with HPLC System

For quantification of major catechins and caffeine, EC, ECG, EGC, EGCG and caffeine were analyzed in the method refer to the paper of Terada *et al.* [33] and partially modified. The peak of catechins and caffeine was detected at 280 nm absorbance with a HPLC system (LC-10A, Shimadzu corporation, Kyoto, Japan) equipped with CAPCELL Pack C18 (UG120S-5, 4.6 × 150 mm, Shiseido). Column oven temperature was at 43 °C. In reference to the previous paper, the tea samples were mixed with triple volume of acetonitrile then filtered with PVDF membrane (Acrodisc LC PVDF, 0.45 µm, 13 mm) quickly. Gradient separation was carried with eluent A (0.1% acetonitrile and 5% *N,N*-dimethylformamide in 0.1% phosphoric acid water solution) and eluent B (acetonitrile). The volume of the injected sample was 10 µL and flow rate was 1.0 mL/min. The gradient program was as follows: Rate of elute B: 0–30 min: 1%→15%; 30–40 min: 15%→90%; and 40–40.1 min: 90%→1%. As the standard, (−)-epigallocatechin, epicatechin, (−)-epicatechin gallate (−)-Epigallocatechin Gallate Hydrate, and Caffeine anhydrous were used in the range of 0–500 µg/mL.

### 4.6. Total Polyphenol Concentration

The total phenol of teas was quantified with the Folin–Denis’ reagent assay [34] (refer to our previous paper [35]). Each sample was diluted with purified water (1/5 dilution) and added to the Folin–Denis’ reagent and 10% sodium carbonate solution (Diluted sample: Folin–Denis’ reagent: 10% sodium carbonate solution = 1:1:1). After the solution was placed for 20 min at room temperature, the solution was centrifuged at 10,000 g and room temperature for 1 min. Then, 700 nm absorbance was measured with a multimode plate reader (EnSpire, PerkinElmer, Waltham, MA, USA). (+)-Catechin Hydrate was used as standard in the range 0–1000 μg/mL. The phenol contents of the teas were calculated as catechin equivalents (mg CE/mL).

### 4.7. Chemical Components Analysis with LC-MS/MS System

The chemical components of strong powder tea and strong leaf tea were analyzed with LC-MS/MS system (Maxis 3G, Bruker, MA, USA) equipped with ACQUITY UPLC BEH C8 Column (1.7 µm, 2.1 mm × 50 mm, Waters, MA, USA). Column oven temperature was 40 °C. Mass spectra were acquired in ESI negative modes. The tea samples were filtrated with the Acrodisc PVDF membrane filter. The binary gradient 10 mM ammonium formate in water (eluent A) and acetonitrile containing 10 mM ammonium formate (eluent B) in the negative mode was applied with injection volume of 5 μL at a constant flowrate of 0.2 mL/min. The gradient program was as follows: Rate of elute A: 0–10 min: 0%→95%; 10–13 min: 95%; and 13–13.1 min: 95%→0%. The detected molecular masses were found in the Human Metabolome Database [36] using MS Search.

### 4.8. Measurement of SOD-Like Activity

In reference to our previous paper [35] and manufacturers’ protocol, the tea samples (no pretreatment) were diluted in the range of 1–32,000 dilution and analyzed with SOD Assay Kit-WST (Inhibition assay of xanthine oxidase). Inhibition rate (%) was calculated by the ratio of the control (water).

### 4.9. Inhibition of Intracellular ROS in a T Cell Line

Jurkat human leukemic T cell line was used for the measurement of intracellular ROS. Referring to the method by Bass *et al.* [37] and partially modified, 20 µM 2’,7’-dichlorofluorescein diacetate was added to 1.25 × 10^5^ cells/mL of the cells, then incubated at 37 °C for 30 min (5% CO_2_). Next, the tea samples were added to the cells at the 500 times dilution followed by 30-min incubation. After the incubation, 200 µM tert-butyl hydroperoxide was added and incubated for 1 h. Then the fluorescent of intracellular ROS was measured with the Enspire plate reader (Ex 485 nm/Em 535nm). Inhibition rate (%) was calculated by the ratio of control (water).

### 4.10. Data Analysis

All statistical analyses were carried out in triplicate. The mean ± standard deviation (SD) (three measurements) was presented. Statistical significance was calculated in Bonferroni–Dunn multiple comparison test or Welch’s *t* test with the Microsoft Office Excel 2013 (Microsoft, Redmond, WA, USA) and the add-in software Statcel 3 (OMS publishing Inc., Saitama, Japan). Significance level was set at 5%.

## 5. Conclusions

In conclusion, the powdering process of green tealeaves with the ceramic mill increased the extracted catechin concentration, especially that of EGCG. Moreover, these powdered teas showed a tendency of higher inhibitory effect of the ROS *in vitro*. Since powdered tea may have different function from leaf tea, further study will be needed.

## Figures and Tables

**Table 1:** Total polyphenol, catechin, and caffeine concentration in green teas by Folin–Denis assay and high-performance liquid chromatography (HPLC) analysis.

	Leaf/Powder Contents (g/80 mL)	Total Polyphenol (μg CE/mL)	EC (μg/mL)	ECG (μg/mL)	EGC (μg/mL)	EGCG (μg/mL)	Caffeine (μg/mL)
Regular	2.00	1275.5 ± 13.91 ^a^	94.5 ± 2.45 ^a^	31.4 ± 3.55 ^a^	354.8 ± 6.9 ^a^	195.6 ± 3.77 ^a^	191.1 ± 1.85 ^a^
Powder	0.36	1121.1 ± 28.96 ^b^	56.3 ± 0.42 ^b^	35.7 ± 5.69 ^a^	191.3 ± 8.75 ^b^	210.6 ± 23.41 ^a^	121.5 ± 1.51 ^b^
Leaf	0.36	373.0 ± 7.20 ^c^	37.0 ± 0.91 ^c^	18.1 ± 1.40 ^a^	38.8 ± 3.14 ^c^	57.9 ± 1.96 ^b^	77.8 ± 0.64 ^c^
Strong Powder	0.96	3055.1 ± 26.35 ^d^	131.1 ± 1.27 ^d^	98.0 ± 21.22 ^b^	513.7 ± 1.74 ^d^	615.1 ± 88.36 ^c^	311.9 ± 4.32 ^d^
Strong Leaf	0.96	1890.9 ± 11.24 ^e^	136.0 ± 0.28 ^e^	33.3 ± 7.67 ^a^	501.2 ± 5.84 ^d^	200.1 ± 6.94 ^a^	244.0 ± 1.88 ^e^
Retention Time (min)	―	―	20.2	30.5	13.0	22.0	10.6

CE: Catechin equivalents. Retention time was calculated by the average of five measurements of standard chemicals. All concentration values represent the mean ± standard deviation (SD) of three measurements. In each column, means with the different superscript letter are significantly different at 5% level (Bonferroni/Dunn method). Regular: Leaf tea steeped without stirring. Powder, Strong Powder: Powdered tea steeped with stirring. Leaf, Strong Leaf: Leaf tea steeped with stirring.

**Table 2:** Differences of catechin area between the Strong Powder tea and Strong Leaf tea measured by liquid chromatography–tandem mass spectrometry (LC-MS/MS) analysis.

Search Compound	Retention Time (min)	[M − H]^−^	Area	
			Strong Powder	Strong Leaf
Catechin/EC	3.9	289.07	2,684,378 ± 89,484.5	3,241,299 ± 192,287.7 *
Gallocatechin/EGC	3.5	305.07	6,418,883 ± 149,286.8 *	6,041,930 ± 16,410.1
Catechin gallate/ECG	4.3	441.08	1,364,591 ± 16,486.5 *	358,874 ± 19,209.7
Gallocatechin gallate/EGCG	3.9	457.08	3,590,762 ± 128,970.1 *	1,043,276 ± 67,495.9

Retention time was calculated by the average of six measurements. Strong Powder and Strong Leaf indicate powdered tea and leaf tea (powder/leaf contents: 0.96 g/80 mL green tea), respectively. All area values represent the mean ± SD of three measurements. * indicates significant difference between Strong Powder and Strong Leaf (Welch’s *t* test, *p* < 0.05).

## Abbreviations

The following abbreviations are used in this manuscript:

AUC	Area under the concentration-time curve
BMI	Body Mass Index
C_max_	Maximum concentration
EC	Epicatechin
ECG	Epicatechin Gallate
EGC	Epigallocatechin
EGCG	Epigallocatechin Gallate
HPLC	High-performance Liquid Chromatography
LC-MS	Liquid Chromatography–Mass Spectrometry
LC-MS/MS	Liquid Chromatography–tandem Mass Spectrometry
ROS	Reactive Oxygen Species
SEM	Scanning Electron Microscope
SD	Standard Deviation
SOD	Superoxide Dismutase
